# Environmental factors and particle size shape the community structure of airborne total and pathogenic bacteria in a university campus

**DOI:** 10.3389/fpubh.2024.1371656

**Published:** 2024-04-08

**Authors:** Tianer Zhang, Mengmeng Liu, Dalin Zhou, Zhijing Ma, Liu Chen, Danchen Wu, Haitao Diao, Wanru Wang, Die Li, Quan Zhen

**Affiliations:** ^1^School of Public Health, Bengbu Medical University, Bengbu, China; ^2^Xinchang Center for Disease Control and Prevention, Shaoxing, China; ^3^Quality Management Department, Fuyang Tumor Hospital, Fuyang, China

**Keywords:** university campus, airborne bacteria, pathogenic bacteria, community structure, indoor, outdoor, particle size, influencing factors

## Abstract

Given the dense population on university campuses, indoor and outdoor airborne bacterial contamination may lead to the rapid spread of diseases in a university environment. However, there are few studies of the characteristics of airborne and pathogenic bacterial communities in different sites on a university campus. In this study, we collected particulate matter samples from indoor and outdoor locations at a university in Bengbu City, Anhui Province, China, and analyzed the community characteristics of airborne and pathogenic bacteria using a high-throughput sequencing technique. The results showed that the composition of the dominant airborne and pathogenic bacterial communities was consistent among sites at the phylum and genus levels, with differences in their relative abundance. There were significant differences in the structure of the airborne and pathogenic bacterial communities between indoor and outdoor sites (*p* < 0.05). An analysis of similarities (ANOSIM) indicated that the structure of airborne bacterial communities in indoor sites was influenced by the room occupancy rate, ventilation conditions, and the extent of indoor furnishing (*p* < 0.05), while the structure of pathogenic bacterial communities was influenced by the number of individuals and spatial dimensions (*p* < 0.05). The impact of particle size on the structure of airborne and pathogenic bacterial communities was relatively minor. A total of 194 suspected pathogenic bacterial species were identified, accounting for 0.0001–1.3923% of the total airborne bacteria, all of which were conditional pathogens. Among them, *Saccharopolyspora rectivirgula*, *Acinetobacter johnsonii*, and *Moraxella osloensis* exhibited relatively high relative abundance, accounting for 24.40, 16.22, and 8.66% of the total pathogenic bacteria, respectively. Moreover, 18 emerging or re-emerging pathogenic bacterial species with significant implications for human health were identified, although their relative abundance was relatively low (0.5098%). The relative abundance of pathogenic bacteria in indoor environments was significantly higher than outdoors, with the laboratory and dormitory having the highest levels. The findings of this study provide valuable guidance for the prevention and control of airborne bacterial contamination and the associated health risks in both a campus environment and other public spaces with high occupancy rates.

## 1 Introduction

The global COVID-19 pandemic has had a severe impact on all aspects of human society, especially health services and the economy. As a result, airborne microorganisms in densely populated public spaces have become a focus of research. Airborne microorganisms are composed of bacteria, fungi, viruses, etc. (1), although bacteria comprise the highest proportion, accounting for about 80% of the total (2). Some bacteria are pathogenic, and may be the vectors of various human diseases. For example, *Legionella pneumophila* and *Streptococcus pneumonia* can invade the human body and cause severe pneumonia (3, 4), while *Staphylococcus aureus*, *Pseudomonas aeruginosa*, and *Acinetobacter baumannii* can cause abscesses, infections, and other diseases (4–6).

The dense population on university campuses and the potential spread of airborne bacterial contamination in both indoor and outdoor environments may lead to the rapid transmission of diseases. Several studies have evaluated the levels of airborne bacteria inside and outside of classrooms (7–9). Some studies have investigated other campus locations, such as libraries (10, 11), dormitory rooms (12), and laboratories (13, 14). However, most studies have focused on a single type of environment. Due to continual improvements to campus facilities in many universities, the types of indoor and outdoor environments on campuses are extremely varied. However, few studies have taken these diverse environments into account (15, 16).

Studies of airborne bacteria in university environments have mainly been conducted by culture methods (17, 18), which provide limited information compared to the use of high-throughput sequencing (HTS). This is because HTS methods provide a long sampling time, ensuring that sufficient amounts of DNA can be extracted, which is not conducive to non-fixed point sampling. Culture methods can detect only 0.1–10% of the total airborne bacteria with a focus on concentration and size distributions (19). Although researchers have selected colonies of viable bacteria and identified the different species, the descriptions of community structure are still incomplete (15, 20, 21). Therefore, there are still many unknown aspects of the bacterial community structure on a university campus, especially the pathogens that are present. This needs to be further investigated through HTS.

Studies in offices and residential areas have shown that the airborne bacterial communities in indoor and outdoor areas differ greatly, and the characteristics of the airborne bacterial communities are affected by human activities, air circulation, and other factors (22–25). We hypothesized that the community structure of airborne and pathogenic bacteria in indoor locations on a university campus is affected by the room occupancy rate, spatial dimensions, air circulation, and the extent of furnishing, while the community structure of airborne and pathogenic bacteria in outdoor locations is affected by their occupancy rate and degree of greening. In this study, we selected nine typical locations—five indoor sites and four outdoor sites—to study the diversity of total airborne bacteria and the relative abundance of pathogenic bacteria in different locations using HTS technology. We also compared the differences in the structure of airborne bacterial communities in the different environments. Considering the differences in human respiratory deposition sites for different particle sizes, we also hypothesized that the campus airborne and pathogenic bacterial community structures are influenced by particle size. To test this hypothesis, our study considered three particle size classes: total suspended particulates (TSP), inhalable particulate matter (PM_10_), and fine particulate matter (PM_2.5_). The results of this study will ensure a more comprehensive understanding of the status of the bacterial community in campus air and provide guidance for protecting the health of students and teachers. The study also provides a reference for the prevention and control of bacteria-related health hazards in other public spaces with high occupancy rates.

## 2 Materials and methods

### 2.1 Overview of the study sites

Sampling sites were established in five indoor locations (dormitory, laboratory, library, canteen, and classroom) and four outdoor locations (basketball court, playground, meadow, and grove) at Bengbu Medical University in Anhui Province, China. The environmental characteristics of each site are presented in Table 1. Besides, a detailed schematic diagram of each sampling point is provided in Supplementary Figure S1, and the layouts of the five indoor locations were displayed in Supplementary Figure S2.

**Table 1:** Environmental characteristics of each sampling site.

Type	Sites	Environment characteristic
Indoor	Dormitory	Fewer people, smaller space, general air circulation, more household goods
	Laboratory	Fewer people, smaller space, better air circulation, more experimental equipment
	Classroom	More people, smaller space, better air circulation, less furnishings
	Library	More people, larger space, general air circulation, many books in the collection
	Canteen	More people, larger space, general air circulation, less furnishings
Outdoor	Basketball court	Concrete floor, surrounded by plants, large circulation of people
	Playground	Plastic floor, surrounded by plants, large circulation of people
	Meadow	Large proportion of vegetation cover, low human entry
	Grove	Large vegetation cover, many trees, and low personnel access

### 2.2 Sample collection

In April 2022, under clear weather conditions, TSP, PM_10_, and PM_2.5_ samples were collected at the nine different sites, resulting in a total of 27 samples (Supplementary Table S1). Sample collection was achieved using a medium-flow particulate sampler (Lao Ying Model 2030, Qingdao Laoshan Applied Technology Research Institute, China) at the height of the breathing zone (1.5 m above the ground) (26), with a continuous flow rate of 100 L/min for 6 h (9:00 to 15:00). Each sample was collected from a volume of approximately 36 m^3^ of air. Airborne aerosols were collected using glass fiber filters (Pall, United States). Prior to sampling, the filters were sealed with aluminum foil and subjected to high-temperature calcination at 450°C for 4 h to prevent microbial contamination. After sampling, the filters were sealed in sterilized aluminum foil and promptly transported back to the laboratory, where they were stored in a freezer at −20°C.

### 2.3 Extraction of DNA and polymerase chain reaction amplification

Each filter was cut into strips and washed by refrigerated sterile 1 × PBS buffer. The elution was filtered using a 0.2 μm Supor 200 PES Membrane Filter (Pall, United States), which was then cut up for DNA ex-traction using the PowerSoil Pro Kit DNA extraction kit (QIAGEN, Germany). The sample DNA was used as the template for the PCR to amplify the 16S rRNA gene using 341F and 806R as primers. The amplified PCR products were subjected to gel excision and target band purification, followed by the construction of a library by pooling the PCR products at equal concentrations. Subsequently, the library was quantified using Qubit and underwent a library quality assessment, ultimately culminating in paired-end sequencing on the HiSeq 2,500 sequencing platform.

### 2.4 High throughput data collation

The total airborne bacteria were identified. After concatenating the initial sequences and removing redundant sequences and chimeras, the sequences were aligned against reference sequences using the Silva database in the Mothur software. This alignment was then subjected to pre-clustering and taxonomic annotation, with operational taxonomic units (OTUs) defined based on a 97% sequence similarity threshold. Subsequently, further annotation was performed in the Qiime software to assign taxonomic information at various levels, including phylum, class, order, family, and genus, based on the sequence similarities.

The airborne pathogenic bacteria were identified. The high-throughput sequences, after excluding redundant sequences and chimeras, were subjected to a local BLASTn analysis using the National Center for Biotechnology Information (NCBI) 16S microbial database as the reference sequence. The resulting geninfo identifier (GI) numbers were matched with the “gitaxidnucl.dmp” file to obtain accurate annotation information. Sequences with a sequence homology greater than 97%, coverage above 95%, and an E-value <1e^−10^ were considered to be successfully identified. Based on the compilation of human pathogenic bacteria provided by the Edinburgh Infectious Diseases Center (27), a total of 538 species of human pathogenic bacteria were collected, including 54 emerging or re-emerging human pathogenic bacteria that pose significant threats to human health. From the identified sequences, pathogenic bacteria sequences with consistent genus and species information were selected to analyze the composition of suspected pathogens and the community structure in each sample.

### 2.5 Statistical analyses

Data processing and statistical analysis were performed using R software. The “vegan” package was used to calculate the number of OTUs and conduct an analysis of similarities (ANOSIM) test for bacterial community analysis. Principal component analysis (PCA) was used to determine the relationships among bacterial communities in different sites. Furthermore, *t*-tests and a one-way ANOVA were conducted on the relevant data. A *p*-value <0.05 was defined as being significantly different.

## 3 Results

### 3.1 Number of OTUs for total airborne bacteria

A total of 1,521,667 valid sequences of the 16S rRNA gene and 18,231 OTUs were obtained. To evaluate the bacterial biodiversity at the same sequencing depth, we randomly resampled the minimum number of sequences for all samples (Supplementary Figure S3). The rarefaction curves tended to be smooth, indicating that the sequencing depth covered all taxa in the samples.

**Figure 1:**
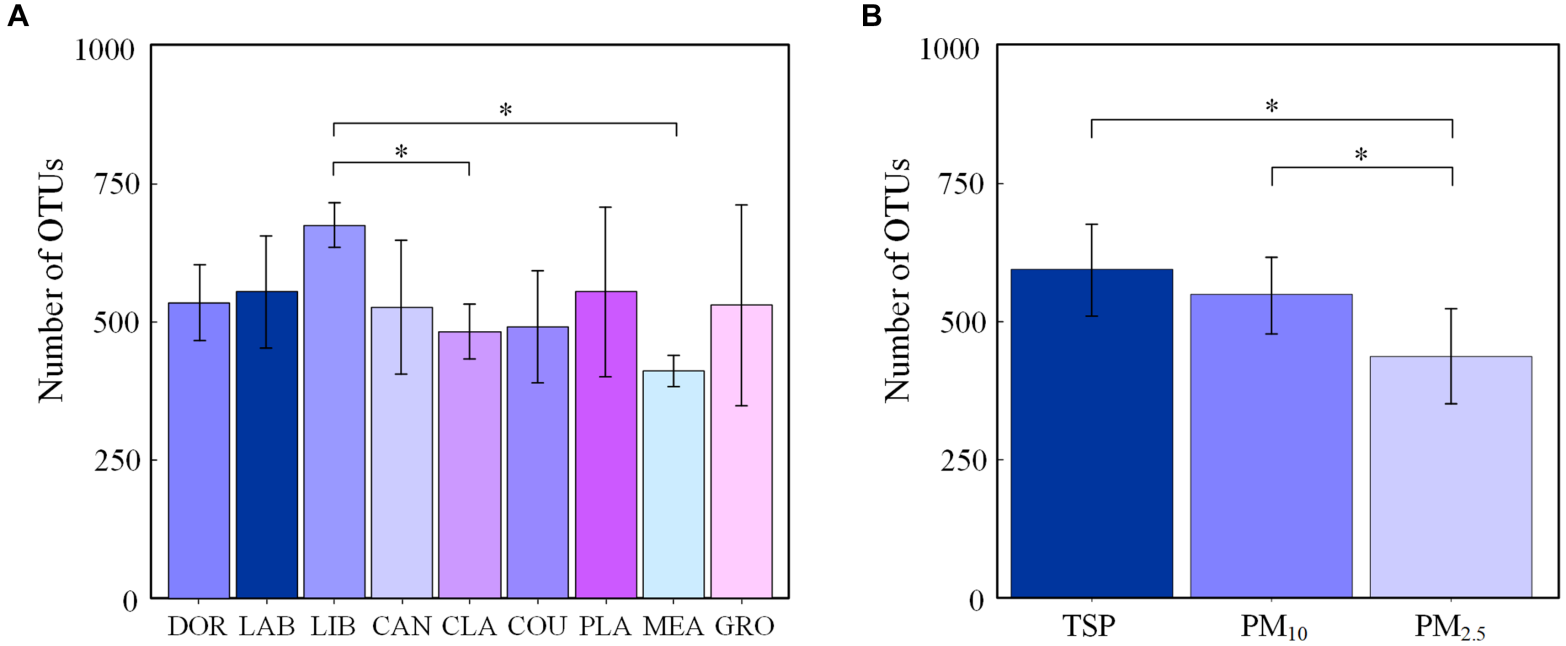
Number of OTUs in airborne bacterial communities at the genus level **(A)** at different sites and **(B)** for different particle sizes (*p < 0.05, 2-tailed). DOR, Dormitory; LAB, Laboratory; LIB, Library; CAN, Canteen; CLA, Classroom; COU, Basketball court; PLA, Playground; MEA, Meadow; GRO, Grove.

The number of OTUs of airborne bacterial communities at different sites and different particle sizes at the genus level is shown in Figure 1, with the highest number of OTUs in the library (675 ± 43) and the lowest number in the classroom and meadow (481 ± 49 and 410 ± 27, respectively). The number of OTUs in the airborne bacterial community was significantly higher in the library than in the classroom and the meadow (*p* < 0.05), while the number of OTUs did not differ significantly among the other sites (*p* > 0.05). There were no significant differences among the groups divided by influencing factors (*p* > 0.05), and the Chao1 index and ACE index exhibited a consistent pattern (Supplementary Table S2). The mean number of airborne bacterial OTUs was 554 for indoor sites and 496 for outdoor sites, with no statistically significant difference (*p* > 0.05). The number of airborne bacterial OTUs was significantly higher in TSP and PM_10_ than in PM_2.5_ (*p* < 0.05), and the Chao1 index and ACE indexes exhibited the same pattern (Supplementary Table S3).

### 3.2 Characterization of the total airborne bacterial community

A total of 38 bacterial phyla and 1707 bacterial genera were detected in this study, and the dominant groups were consistent across sites, with Proteobacteria (46.91%), Actinobacteria (24.61%), Firmicutes (18.45%), and Bacteroidetes (5.61%) accounting for the majority, i.e., >95.58%. *Methylobacterium* (12.37%), *Bradyrhizobium* (6.59%), *Sphingomonas* (5.87%), *Bacillus* (3.60%), and *Streptomyces* (3.56%) were the dominant genera, accounting for >31.99%.

**Figure 2:**
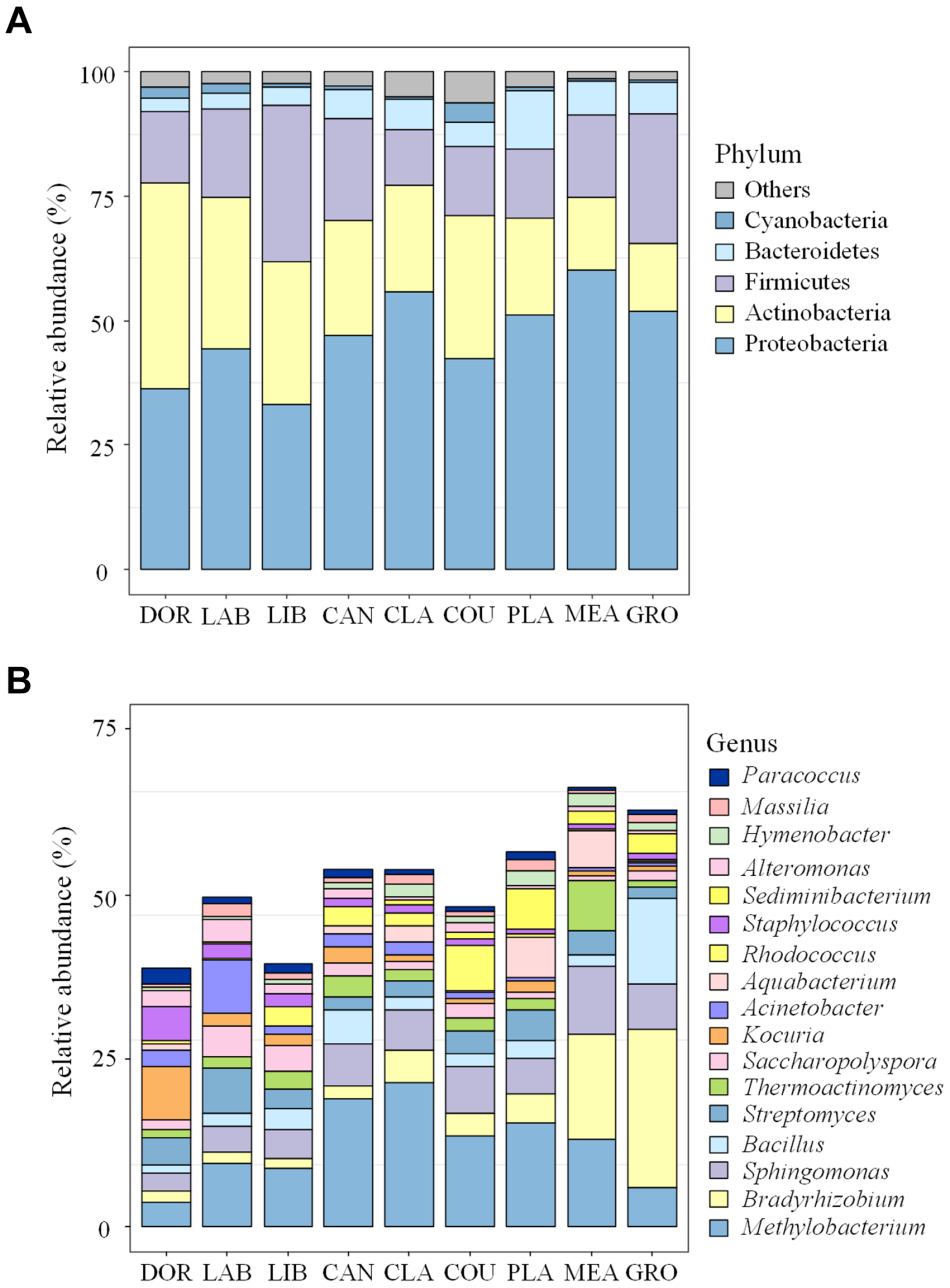
Bacterial community compositions at different sites **(A)** the phylum level; **(B)** the genus level. The term “others” represents all the remaining bacterial phylum/genus. DOR, Dormitory; LAB, Laboratory; LIB, Library; CAN, Canteen; CLA, Classroom; COU, Basketball court; PLA, Playground; MEA, Meadow; GRO, Grove.

Figure 2 shows the dominant groups of airborne bacteria in different sites at the phylum and genus levels. At the phylum level (Figure 2A), Proteobacteria had the highest relative abundance of 60.21% in the meadow and Actinobacteria had the highest relative abundance in the dormitory (41.34%). Firmicutes had the highest relative abundance in the library (31.51%), and accounted for 11.28 to 26.06% of all phyla at the other sites. At the genus level (Figure 2A), *Methylobacterium* had the highest relative abundance in classrooms (21.61%) and *Bradyrhizobium* had the highest relative abundance in groves and meadows at 24.04 and 15.74%, respectively.

**Figure 3:**
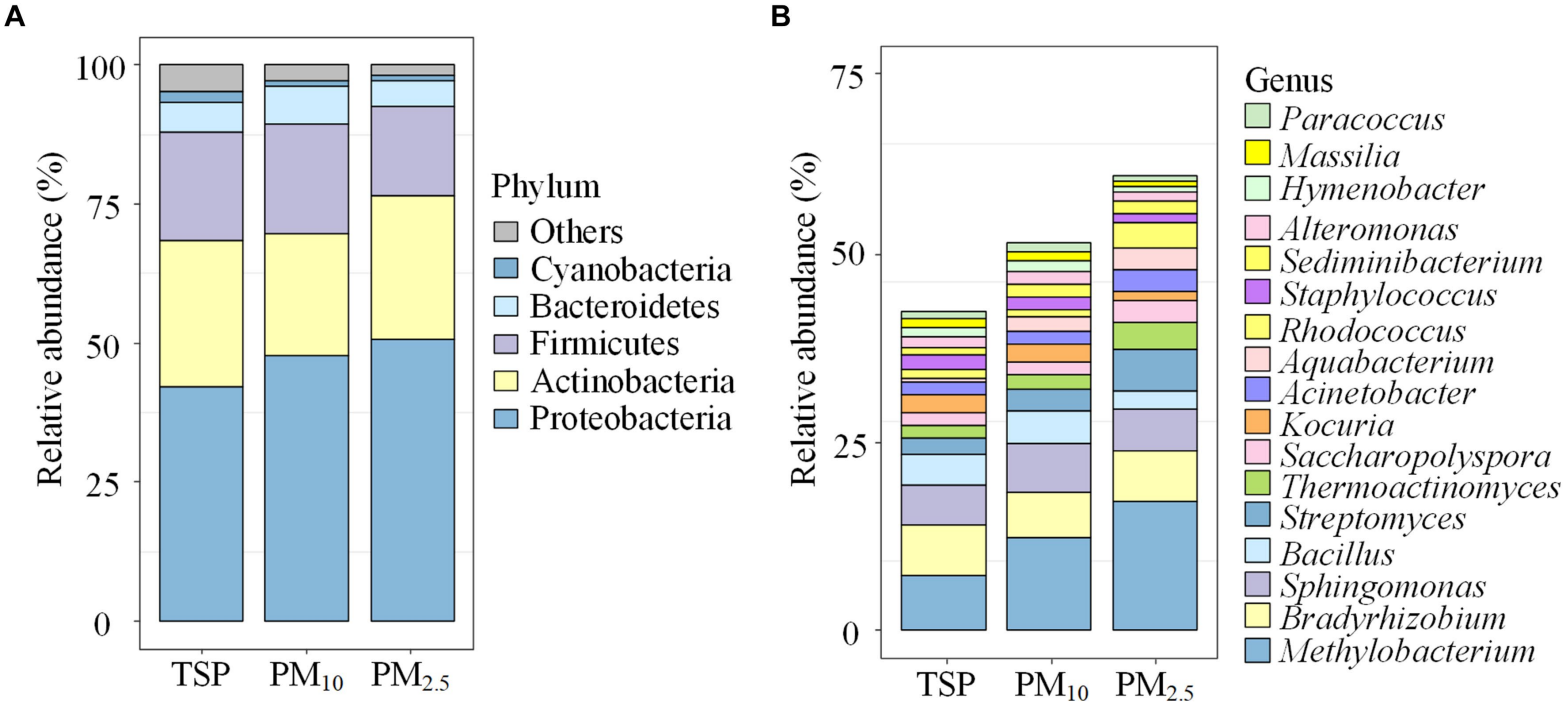
Bacterial community compositions on the different particle sizes: **(A)** the phylum level, and **(B)** the genus level. The term “others” represents the remaining bacterial phyla/genera. DOR, Dormitory; LAB, Laboratory; LIB, Library; CAN, Canteen; CLA, Classroom; COU, Basketball court; PLA, Playground; MEA, Meadow; GRO, Grove.

The community composition of airborne bacteria at the phylum and genus levels on the different particle sizes is shown in Figure 3. At the phylum level (Figure 3A), the relative abundance of Proteobacteria in PM_2.5_ was 50.71%, 1.06 times higher than PM_10_ (47.81%) and 1.20 times higher than TSP (42.21%). Actinobacteria had a relative abundance of 26.15% in TSP, higher than PM_2.5_ (25.85%) and PM_10_ (21.84%). For Firmicutes, the distribution pattern was PM_10_ > TSP > PM_2.5_, with a relative abundance of 19.75, 19.60, and 16.00%, respectively. At the genus level (Figure 3B), the relative abundance of *Methylobacterium* followed the order of PM_2.5_ > PM_10_ > TSP, with values of 17.39, 12.33, and 7.38%, respectively. For *Bradyrhizobium*, the relative abundance followed the order of PM_2.5_ > TSP > PM_10_, with a relative abundance of 6.83, 6.72, and 6.21%, respectively.

**Figure 4:**
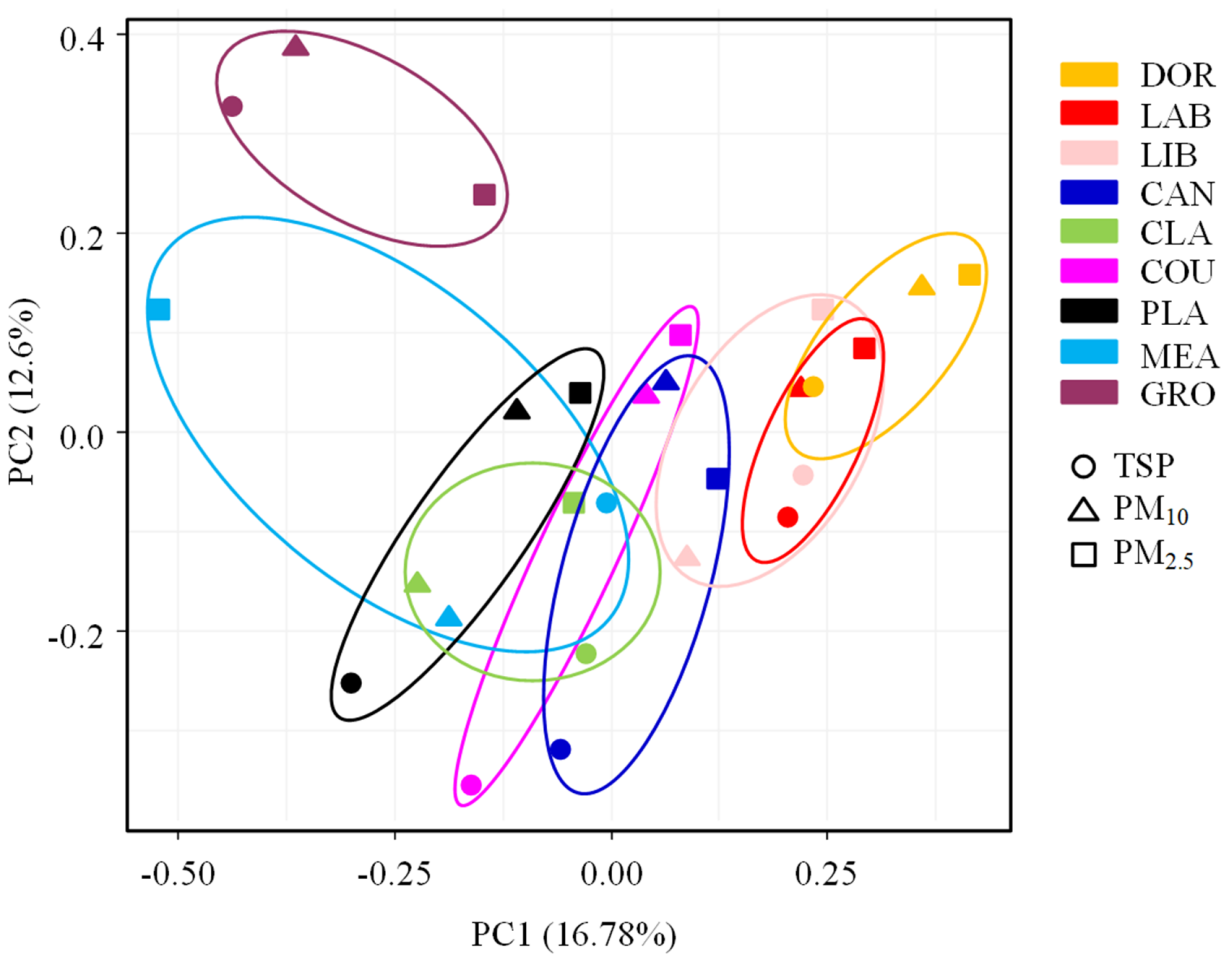
The PCA analysis of airborne bacterial communities at different sampling sites. DOR, Dormitory; LAB, Laboratory; LIB, Library; CAN, Canteen; CLA, Classroom; COU, Basketball court; PLA, Playground; MEA, Meadow; GRO, Grove.

The PCA analysis showed that the bacterial communities differed significantly between sites (Figure 4), with this result also supported by the ANOSIM test (*R* = 0.3649, *p* < 0.05). Consistent with our hypothesis, indoor environments were clearly differentiated from outdoor environments and exhibited specific transitional characteristics. In the indoor environment, the less intensively used dormitory and laboratory were positioned close together on the PCA plot (also supported by the heatmap, Supplementary Figure S4). The well-furnished dormitory, laboratory, and library were close together. The moderately ventilated canteen was clustered with the dormitory, laboratory, and library. Although it was an indoor environment, the classroom was spacious and well ventilated and was clustered with the basketball court and playground in the outdoor environment. The basketball court and playground, and meadow and grove with high levels of vegetation cover were in close proximity.

The ANOSIM test (Supplementary Table S4) revealed that the indoor and outdoor airborne bacterial community structure was significantly different (*R* = 0.2101, *p* < 0.05). The room occupancy rate, air circulation, and the extent of furnishing in indoor sites had a significant effect on the airborne bacterial community structure (*p* < 0.05). The spatial dimensions had no statistically significant effect on the airborne bacterial community structure in indoor sites (*p* > 0.05). There was no statistically significant effect of floor type or the occupancy rate on the community structure in outdoor sites (*p* > 0.05). The airborne bacterial community structure was significantly different among the different particle sizes (*R* = 0.1068, *p* < 0.05), but due to the small *R* value, the differences observed between different sampling sites were more pronounced than the differences observed among particle sizes. The heatmap showed that TSP and PM_10_ were clustered together (Supplementary Figure S5).

### 3.3 Characteristics of airborne pathogenic bacterial communities

The annotated genus-species information obtained by comparing high-throughput sequences using the native BLASTn approach was less extensive than the bacterial community data obtained by a comparison with the Mothur software Silva database (Supplementary Figure S6). The dominant genera obtained by both methods at the family-genus level were generally consistent in terms of taxa and relative abundance (Supplementary Table S5), indicating that the BLASTn analysis was reliable and could be used for the further analysis of airborne pathogenic bacteria.

**Figure 5:**
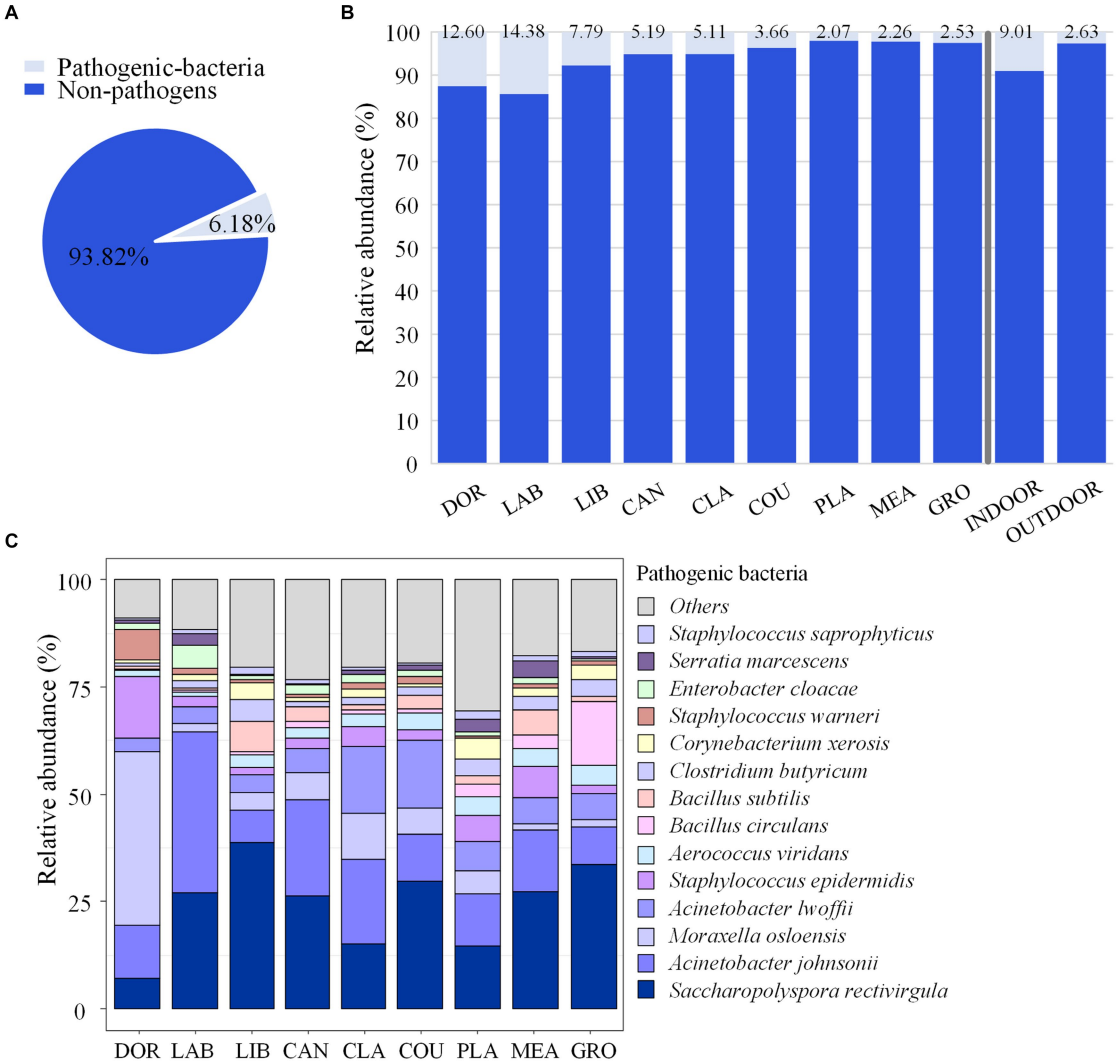
Pathogenic bacterial community composition. **(A)** Relative abundance of pathogenic and non-pathogenic bacteria. **(B)** Relative abundance of pathogenic and non-pathogenic bacteria at each site. **(C)** Community composition of pathogenic bacteria at each site. DOR, Dormitory; LAB, Laboratory; LIB, Library; CAN, Canteen; CLA, Classroom; COU, Basketball court; PLA, Playground; MEA, Meadow; GRO, Grove.

From a catalogue of 538 human pathogenic bacteria, a total of 194 suspected pathogenic bacteria were identified in this study, and their relative abundances accounted for 6.18% of the total airborne bacterial community (Figure 5A). Eighteen species of emerging or re-emerging pathogenic bacteria with a high impact on humans were identified, but in terms of abundance they only accounted for 0.5098% of the total bacteria (Supplementary Table S6).

The highest relative abundance of pathogenic bacteria was found in the laboratory and dormitory at 14.38 and 12.60%, respectively, while the relative abundance of pathogenic bacteria in the other sites ranged from 2.07 to 7.79%, and the relative abundance of pathogenic bacteria in indoor sites accounted for 9.01% of the total bacteria, which was significantly higher than that in the outdoor environment (2.63%, *p* < 0.05), as shown in Figure 5B. The pathogenic bacteria with an average relative abundance >3% were *Saccharopolyspora rectivirgula* (24.40%), *Acinetobacter johnsonii* (16.22%), *Moraxella osloensis* (8.66%), *Acinetobacter lwoffii*, (7.48%), *Staphylococcus epidermidis* (4.81%), and *Aerococcus viridans* (3.08%). The relative abundance of *S. rectivirgula* was highest in the library and grove at 38.72 and 33.72%, respectively, while the relative abundance of *A. johnsonii* was highest in the laboratory, canteen, and classroom at 37.52, 22.49, and 19.58%, respectively. The highest relative abundance *M. osloensis* was in the dormitory at of 40.40% (Figure 5C).

**Figure 6:**
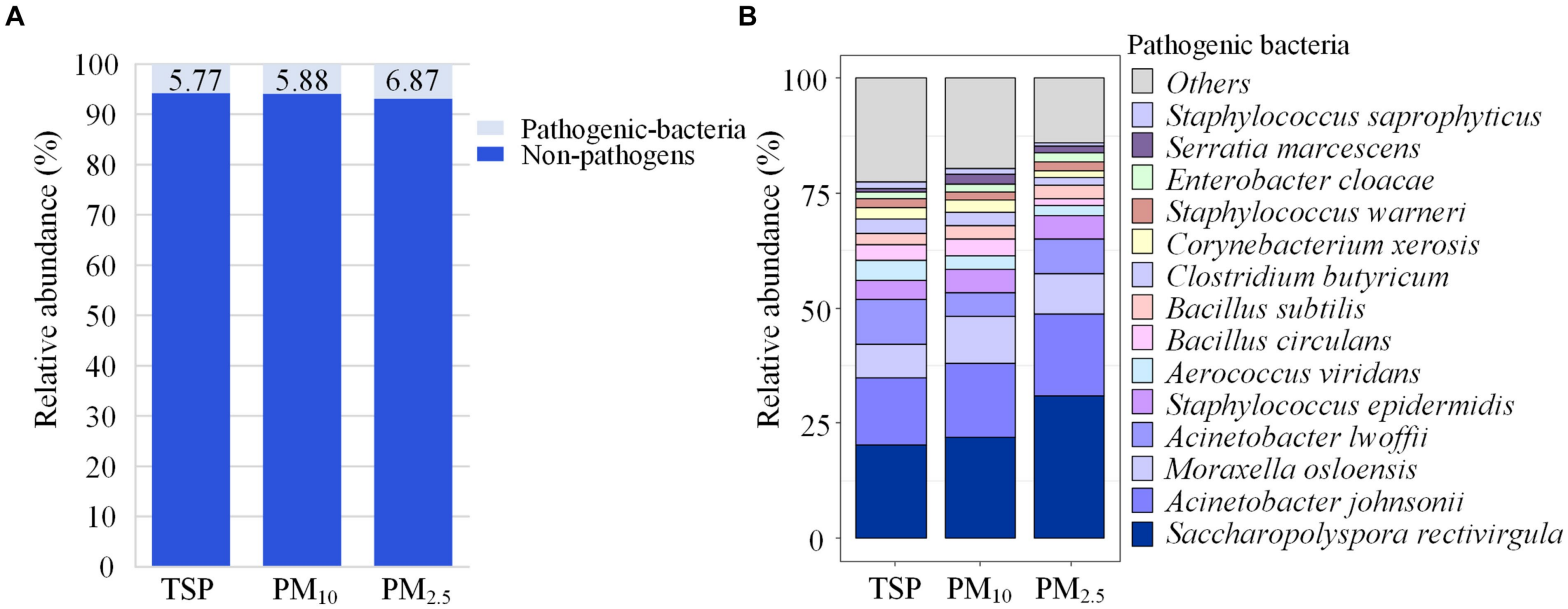
Community composition of pathogenic bacteria. **(A)** Relative abundance of pathogenic and non-pathogenic bacteria in each particle size. **(B)** Community composition of pathogenic bacteria in each particle size. DOR, Dormitory; LAB, Laboratory; LIB, Library; CAN, Canteen; CLA, Classroom; COU, Basketball court; PLA, Playground; MEA, Meadow; GRO, Grove.

Figure 6A shows the proportion of pathogenic bacteria in TSP, PM_10_ and PM_2.5_. The proportion of pathogenic bacteria in PM_2.5_ (6.87%) was slightly higher than that of PM_10_ (5.88%) and TSP (5.77%), but the differences were not significant. *Saccharopolyspora rectivirgula* had a relative abundance of 31.05% in PM_2.5_, higher than PM_10_ (21.98%) and TSP (20.16%), while *Moraxella osloensis* had a relative abundance of 10.02% in PM_10_, higher than PM_2.5_ (8.70%) and TSP (7.26%), as shown in Figure 6B.

The ANOSIM test revealed significant differences in the pathogenic bacterial community structure between sites (*R* = 0.5162, *p* < 0.05), which was consistent with our hypothesis. The PCA analysis also revealed a distinct clustering of pathogenic bacterial communities at different sites, with the smallest site (dormitory) being positioned on the PCA plot far from the other sites (also supported by the heatmap, Supplementary Figure S7). The more crowded and spacious library, canteen, and classroom were clustered together. The classroom, which was a spacious environment with good air circulation, was partially clustered with some outdoor sites (i.e., basketball court and playground). Pathogenic bacterial communities from outdoor sites were clustered together, indicating a high degree of similarity.

**Figure 7:**
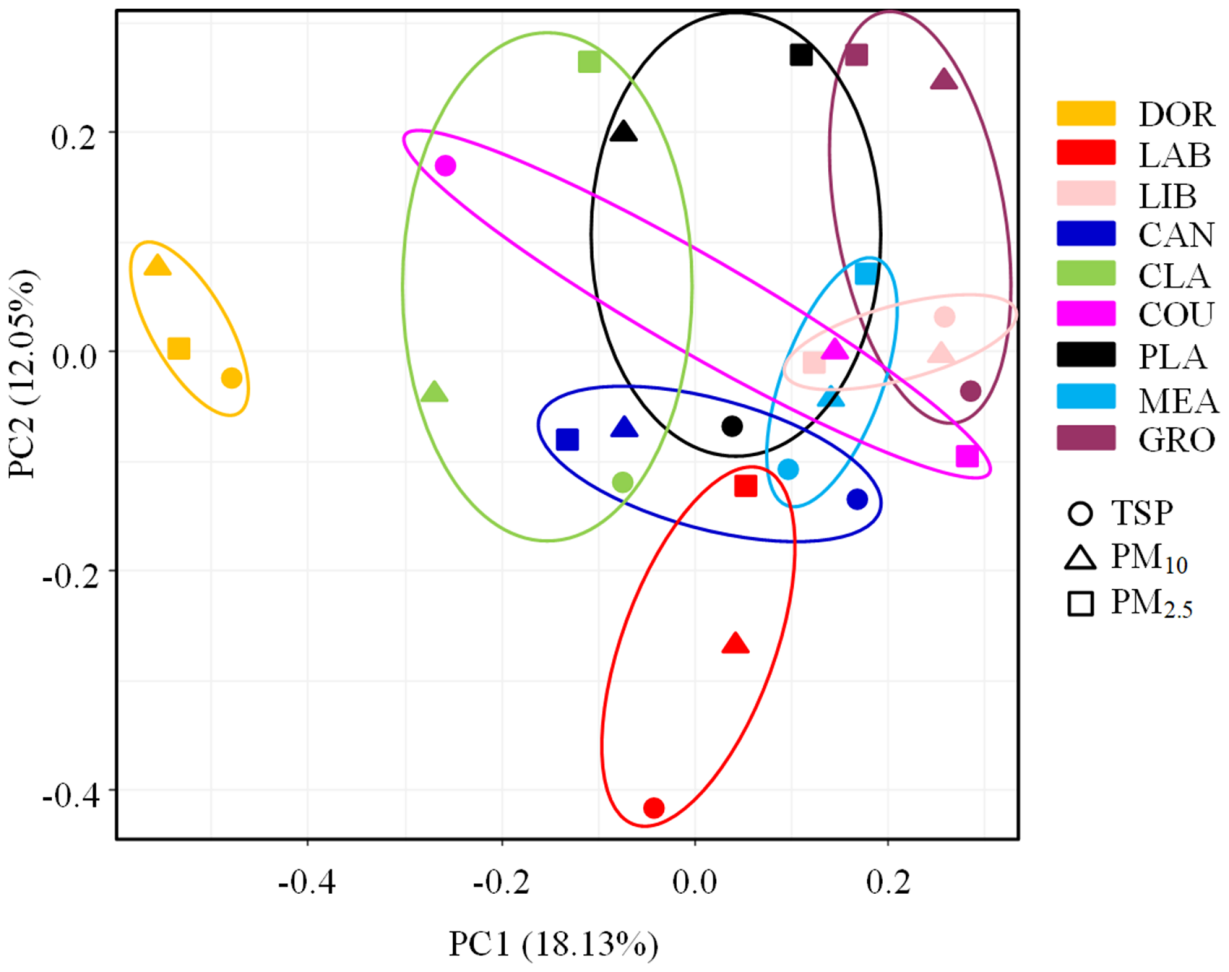
PCA analysis of pathogenic bacterial communities at different sampling sites. DOR, Dormitory; LAB, Laboratory; LIB, Library; CAN, Canteen; CLA, Classroom; COU, Basketball court; PLA, Playground; MEA, Meadow; GRO, Grove.

According to the ANOSIM test (Supplementary Table S7), there was a significant difference in the community structure of airborne pathogenic bacteria between the indoor and outdoor environments (*R* = 0.2396, *p* < 0.05), the room occupancy rate and size of indoor locations had a significant effect on the airborne pathogenic bacterial community structure (*p* < 0.05). The air circulation conditions and the extent of furnishing had no statistically significant effect on the airborne pathogenic bacterial community structure in indoor locations (*p* > 0.05), and the floor type and occupancy rate had no statistically significant effect on community structure in outdoor locations (*p* > 0.05). The differences in community structure of airborne bacteria across the different particle sizes were not statistically significant (*R* = 0.0319, *p* > 0.05, Supplementary Figure S8), suggesting that the effect of location on the community structure of airborne pathogenic bacteria outweighed that of particle size, as shown in Figure 7.

## 4 Discussion

### 4.1 Number of total airborne bacteria OTUs

The total airborne bacterial OTUs were determined at the different sites. There were no significant differences in airborne bacterial community species richness among the sites except for the significant differences between the library, meadow, and classroom, which was inconsistent with our hypothesis. The similar number of OTUs at various sites may be related to indoor–outdoor gas circulation, and air exchange between different sites through window ventilation and door penetration, resulting in a similar species richness of indoor and outdoor airborne bacterial communities (7, 28). The higher species richness of airborne bacterial communities in the library may be related to the large book collections and bacterial growth in books left standing for long periods of time (29). The low species richness in the meadow may be related to the bactericidal effect of plants (30, 31), while the low species richness in classrooms may be related to their regular disinfection during the COVID-19 pandemic.

The number of total airborne bacterial OTUs was higher in TSP than PM_10_ and PM_2.5_. Lu et al. (32) found that airborne bacterial species richness in atmospheric particles followed the order of TSP > PM_10_ > PM_2.5_. Bowers (33) et al. found that bacterial species richness was higher in coarse particles than fine particles. The results of this study were consistent with these observations and considered logical given that PM_10_ and PM_2.5_ are fractions of TSP, and bacteria commonly attach to coarse particles. Coarse particles have a high shaded surface area, which can protect bacteria from UV radiation, and are also carbon and energy sources, providing abundant nutrients. Coarse particles are therefore favorable environments for bacterial survival growth and reproduction (32).

### 4.2 Characterization of the total airborne bacterial communities

We found that different locations and particle sizes exhibited similar dominant airborne bacterial compositions at the phylum and genus levels. At the phylum level, the dominant bacterial phyla were Proteobacteria, Actinobacteria, Firmicutes, and Bacteroidetes, which agreed with the findings of Chen et al. (34) at a Wuhan university campus and Sun et al. (35) at a nursery in Taiyuan. At the genus level, the dominant bacterial genera were *Methylobacterium*, *Bradyrhizobium*, *Sphingomonas*, *Bacillus*, and *Streptomyces*, which was consistent with research conducted at multiple sites (24, 36–39). This consistency may reflect the widespread natural distribution of these bacteria and their strong adaptability to the atmospheric environment.

In accordance with our hypothesis, the ANOSIM test revealed significant differences in the airborne bacterial community structures of indoor and outdoor air at sampling sites around a university campus, with each site exhibiting transitional characteristics. Chen et al. (34) found significant differences in the airborne bacterial community structure between indoor and outdoor locations at Wuhan University of Technology, and the results of the present study were consistent with this finding. Human activities are the primary sources of bacteria in indoor air, with microorganisms entering indoor air through direct shedding from skin and clothing surfaces, the resuspension of settled particles caused by human movement, direct or indirect contact with indoor surfaces, and emissions via exhalation (9). Humans are estimated to emit approximately 14 million bacterial cells and 14 million spores per hour, resulting in an 80-fold higher airborne bacteria number compared to a control environment (40). In contrast, the bacterial sources in outdoor air are primarily from natural elements such as soil, water bodies, and plants, with fewer human activities and better air circulation in outdoor environments compared to indoors. These factors may account for the differences observed in the airborne bacterial community structures between indoor and outdoor air.

The ANOSIM test found that the room occupancy rate, air circulation, and the extent of furnishing were factors affecting the bacterial community structure in indoor locations, which was consistent with our hypothesis. The results of the PCA analysis also confirmed our hypothesis. Contrary to our expectation, the effect of the spatial dimensions of an indoor location on the bacterial community structure was not statistically significant. The dormitory, laboratory, and library were relatively close together on the PCA plot, and the extent of furnishing was the main factor influencing the differences in bacterial community structure in indoor places. There were many living organisms in the dormitory, a large number of scientific research instruments and experimental equipment in the laboratory, and a large collection of books and documents in the library. In addition, the extent of furnishing may affect cleaning methods and maintenance and handling conditions, which might alter the community structure of airborne bacteria. Ventilation conditions also played a role in shaping the airborne bacterial community structure in indoor environments (41). Wang et al. (42) investigated the impact of air exchange rates on indoor bacterial concentrations, and found that higher air exchange rates could mitigate the influence of occupant density on indoor bacterial concentration, indicating the strong influence of ventilation conditions on airborne bacteria. The windows in the dormitory are only opened infrequently, resulting in relatively poor ventilation. The library is a large area with windows installed only on one side, leading to inadequate ventilation and poor air movement. Similarly, although the canteen is a large area, the doors and windows are not often open, resulting in poor air circulation. The room occupancy rate was a factor influencing the airborne bacterial structure in indoor environments. This was due to the dust generated by people moving around, as well as the release of bacteria through shedding from the skin, coughing, and sneezing, which contributed to the differences observed in airborne bacterial community structure.

The relative abundance of *Bradyrhizobium* in the meadow and grove was higher than in other sites. This genus primarily exists within the soil microbial community and typically colonizes the roots of plants, capturing atmospheric nitrogen to provide nutrients for vegetation (36). The extensive soil and vegetation cover at the meadow and grove sample sites could explain the higher relative abundance of *Bradyrhizobium* compared to the indoor environments. However, the ANOSIM test indicated that this factor did not significantly influence the differences in the airborne bacterial community structure in the outdoor environment, which was inconsistent with our hypothesis. The type of ground surface and occupancy rate of outdoor sites had a minimal impact on the structure of their airborne bacterial communities. This was attributed to the strong air circulation in outdoor environments, the proximity of the different sites, and the exchange and movement of bacteria via air masses.

### 4.3 Characteristics of airborne pathogenic bacterial communities

Airborne transmission of pathogenic bacteria in the atmosphere may pose potential threats to human health. The airborne pathogenic bacteria identified in this study were opportunistic pathogens that generally do not cause harm to the human body. However, they do pose a risk to immunocompromised individuals, such as post-operative patients, leading to various infections and diseases, including allergies, wound infections, and respiratory tract illnesses. Supplementary Table S8 provides a list of the sources and potential health hazards of the six dominant pathogenic bacteria, whose relative abundance exceeded 3%.

The relative abundance of pathogenic bacteria in indoor sites was significantly higher than in outdoor sites, with the highest abundance found in the laboratory and dormitory, which may be due to the large number of scientific research instruments and supplies in the laboratory. Because the instruments are only occasionally moved, they form sanitary dead corners and are prone to the accumulation of dust. The dormitory is a small room with items such as bedclothes where bacteria can easily breed. There is also an adjoining bathroom where bacteria can easily breed. Indoor environmental hygiene is mainly dependent on cleaning by staff and the frequency of opening windows for ventilation. The typically low frequency of cleaning by students in dormitories and tendency to keep doors closed may have led to the relatively high abundance of pathogenic bacteria in the dormitory. Conversely, outdoor areas benefit from better air circulation and the potential antimicrobial properties of volatile organic compounds emitted by plants (31). Therefore, the frequency of cleaning of indoor sites should be strengthened, especially laboratories and dormitories.

The ANOSIM test revealed a significant difference in the airborne bacterial community structure between indoor and outdoor environments, which confirmed our hypothesis. The PCA analysis corroborated this finding. The room occupancy rate influenced the indoor airborne bacterial community structure, possibly due to factors such as the presence of resident bacteria on human skin surfaces, including *S. epidermidis*, *Micrococcus*, *S. aureus*, and *Streptococcus*, as well as the release of bacteria from bodily secretions (e.g., sweat), and the settling of dust particles caused by human activities. In contrast, outdoor environments exhibited a broader range of sources for pathogenic bacteria and benefited from better air circulation (40, 43–45).

The room occupancy rate and size of indoor spaces were factors that influenced the differences in the pathogenic bacterial community structure. This finding was consistent with our hypothesis. However, contrary to our expectations, the impact of air circulation and the extent of furnishing on the structure of airborne pathogenic bacteria communities was relatively small. This suggests that air circulation conditions and the extent of furnishing were not the primary factors influencing the structure of pathogenic bacteria communities. There was a significant difference in the community structure between the small dormitory and other sites. The relative abundance of *M. osloensis* in the dormitory was significantly higher than at other sites. *Moraxella osloensis* is found in several locations in the indoor environment, such as sinks and laundry rooms (46). Dormitories, with their high occupancy rate and the lack of regular cleaning and disinfection of items like bed sheets, blankets, and daily necessities, may serve as breeding grounds for *M. osloensis*. The influence of the different ground surfaces in outdoor locations and the number of individuals on the structure of airborne pathogenic bacteria communities was relatively small, possibly due to the strong air circulation in the outdoor environments that facilitated airborne bacterial exchange between the meadow, grove, basketball court, and playground.

We detected 18 emerging or re-emerging pathogenic bacteria, which are defined by the Centers for Disease Control and Prevention as “infectious diseases that have shown an increased incidence in humans over the past 20 years or have the potential to increase in the near future” (47). In this study, the most abundant emerging or re-emerging pathogenic bacteria detected in samples were *S. epidermidis* (0.3408%), *Serratia marcescens* (0.0843%), and *Corynebacterium amycolatum* (0.0288%). *Staphylococcus epidermidis* is commonly found on human skin, mucous membranes, and in the environment. It is a common hospital pathogen that poses a threat to immunocompromised patients, causing conditions such as pyogenic infections and urinary tract infections. *Serratia marcescens* is present in soil, water, and plants, and it is associated with urinary tract infections (48, 49), respiratory tract infections, and other diseases (50). *Corynebacter amycolatum* can be isolated from urine and sputum, and it can cause ear infections (51). Additionally, we detected small numbers of *S. aureus*, *Klebsiella pneumoniae*, *P. aeruginosa*, *Haemophilus influenzae*, *Campylobacter jejuni*, and other bacteria that present health risks that should not be overlooked.

## 5 Conclusion

By analyzing the characteristics of airborne and pathogenic bacterial communities at different locations and in different particle sizes on a university campus, this study revealed significant differences in the airborne and pathogenic bacterial communities between indoor and outdoor environments. Particle size had a relatively minor impact on their structure. Factors such as the room occupancy rate, ventilation conditions, and the extent of furnishing in indoor sites influenced the structure of the bacterial communities, while the number of individuals and spatial dimensions of rooms specifically affected the structure of the airborne pathogenic bacterial communities. The relative abundance of pathogenic bacteria in indoor environments was significantly higher than in outdoor environments, with the highest levels observed in a laboratory and dormitory. This highlights the need for universities to promote hygiene awareness, establish regular cleaning and disinfection protocols, encourage students to develop good hygiene habits, and improve indoor air quality by increasing ventilation frequency, among other measures. These measures will improve the health of both teachers and students.

## Data availability statement

The original contributions presented in the study are publicly available. This data can be found in NCBI SRA online repositories under BioProject accession number PRJNA1064884.

## Author contributions

TZ: Writing – original draft, Conceptualization, Data curation, Formal analysis, Methodology, Validation. ML: Formal analysis, Validation, Writing – review & editing. DZ: Formal analysis, Project administration, Resources, Writing – original draft. ZM: Formal analysis, Project administration, Resources, Writing – original draft. LC: Investigation, Resources, Validation, Writing – review & editing. DW: Investigation, Resources, Validation, Writing – review & editing. HD: Resources, Visualization, Writing – review & editing. WW: Resources, Visualization, Writing – review & editing. DL: Resources, Visualization, Writing – review & editing. QZ: Conceptualization, Funding acquisition, Methodology, Project administration, Software, Supervision, Writing – review & editing.
