# Supplementary material for: Environmental factors and particle size shape the community structure of airborne total and pathogenic bacteria in a university campus
Source: Front Public Health. 2024 Apr 8;12:1371656. doi: 10.3389/fpubh.2024.1371656 (PMC11033423; doi:10.3389/fpubh.2024.1371656)
Supplement: Supplementary file 1 [file Data_Sheet_1.docx]

Supplementary Material

**Table S1.** The abbreviation of each sample collection.

| Type | Sampling Site | Abbreviation | Partical size | | |
| --- | --- | --- | --- | --- | --- |
|  |  |  | TSP | PM_10_ | PM_2.5_ |
| Indoor | Dormitory | DOR | TDOR | IDOR | FDOR |
|  | Laboratory | LAB | TLAB | ILAB | FLAB |
|  | Library | LIB | TLIB | ILIB | FLIB |
|  | Canteen | CAN | TCAN | ICAN | FCAN |
|  | Classroom | CLA | TCLA | ICLA | FCLA |
| Outdoor | Basketball court | COU | TCOU | ICOU | FCOU |
|  | Playground | PLA | TPLA | IPLA | FPLA |
|  | Meadow | MEA | TMEA | IMEA | FMEA |
|  | Grove | GRO | TGRO | IGRO | FGRO |

**Table S2.** Alpha-diversity of airborne bacterial communities of different sites at the genus level.

| Group | No. of OTUs | Chao1 index | ACE index | Pielou index | Shannon index | Simpson index |
| --- | --- | --- | --- | --- | --- | --- |
| DOR | 535±69 | 672.80±114.87 | 673.39±128.22 | 0.71±0.00 | 6.39±0.10 | 0.97±0.00 |
| LAB | 555±100 | 673.26±118.28 | 676.13±111.01 | 0.70±0.07 | 6.37±0.81 | 0.96±0.03 |
| LIB | 675±43 | 831.15±15.45 | 830.79±44.10 | 0.74±0.03 | 6.95±0.32 | 0.98±0.01 |
| CAN | 526±122 | 686.49±126.45 | 683.72±124.25 | 0.66±0.10 | 5.96±1.16 | 0.93±0.08 |
| CLA | 481±49 | 573.68±38.06 | 568.62±14.37 | 0.67±0.13 | 5.95±1.28 | 0.92±0.07 |
| COU | 491±106 | 654.33±142.50 | 644.10±156.25 | 0.59±0.08 | 5.30±0.93 | 0.90±0.06 |
| PLA | 554±157 | 711.92±172.85 | 707.80±186.18 | 0.66±0.10 | 6.04±1.15 | 0.94±0.05 |
| MEA | 410±27 | 549.92±15.03 | 546.40±55.97 | 0.58±0.11 | 5.00±1.02 | 0.89±0.08 |
| GRO | 530±183 | 657.33±151.96 | 622.02±173.18 | 0.71±0.15 | 6.39±1.70 | 0.94±0.08 |

Values are presented as the means ± SD.

**Table S3.** Alpha-diversity of airborne bacterial communities of different sizes at the genus level.

| Partical size | No. of OTUs | Chao1 index | ACE index | Pielou index | Shannon index | Simpson index |
| --- | --- | --- | --- | --- | --- | --- |
| TSP | 596±95^a^ | 747.75±104.85^a^ | 750.23±110.66^a^ | 0.71±0.11^a^ | 6.56±1.08^a^ | 0.96±0.06 |
| PM_10_ | 551±84^a^ | 689.15±94.23^a^ | 685.11±78.05^a^ | 0.67±0.08^ab^ | 6.11±0.89^ab^ | 0.94±0.05 |
| PM_2.5_ | 438±101^b^ | 566.73±100.25^b^ | 548.99±115.53^b^ | 0.62±0.09^b^ | 5.44±0.92^b^ | 0.91±0.06 |

Values are presented as the means ± SD.

Different letters in a column indicate a statistically significant difference at *P* < 0.05 between samples.

**Table S4.** The ANOSIM test of total airborne bacterial communities between different influencing factors.

| Type | Factors | *R* | *P* |
| --- | --- | --- | --- |
| Indoor | People | 0.1939 | **0.042** |
|  | Space | 0.1089 | 0.111 |
|  | Ventilation condition | 0.5161 | **0.021** |
|  | Furnishings | 0.3704 | **0.015** |
| Outdoor | Ground type | 0.0704 | 0.216 |
|  | People | 0.0704 | 0.224 |

**Table S5.** Comparison of the results of Blastn and high-throughput sequencing analysis.

| Analyzed by Mothur at class-genus level | Relative abundance (%) | Analyzed by Blastn at class-genus level | Relative abundance (%) |
| --- | --- | --- | --- |
| *Methylobacteriaceae_Methylobacterium* | 12.37 | *Methylobacteriaceae_Methylobacterium* | 15.24 |
| *Bradyrhizobiaceae_Bradyrhizobium* | 6.59 | *Sphingomonadaceae_Sphingomonas* | 6.98 |
| *Sphingomonadaceae_Sphingomonas* | 5.87 | *Bradyrhizobiaceae_Bradyrhizobium* | 6.14 |
| *Bacillaceae_Bacillus* | 3.60 | *Bacillaceae_Bacillus* | 4.81 |
| *Streptomycetaceae_Streptomyces* | 3.56 | *Streptomycetaceae_Streptomyces* | 4.31 |
| *Thermoactinomycetaceae_Thermoactinomyces* | 2.47 | *Thermoactinomycetaceae_Thermoactinomyces* | 2.85 |
| *Pseudonocardiaceae_Saccharopolyspora* | 2.10 | *Moraxellaceae_Acinetobacter* | 2.30 |
| *Micrococcaceae_Kocuria* | 2.07 | *Burkholderiales__Aquabacterium* | 2.17 |
| *Moraxellaceae_Acinetobacter* | 2.01 | *Pseudonocardiaceae_Saccharopolyspora* | 2.14 |
| *Comamonadaceae_Aquabacterium* | 1.87 | *Micrococcaceae_Kocuria* | 2.14 |
| *Nocardiaceae_Rhodococcus* | 1.84 | *Staphylococcaceae_Staphylococcus* | 1.93 |
| *Staphylococcaceae_Staphylococcus* | 1.63 | *Clostridiaceae_Clostridium* | 1.72 |
| *Chitinophagaceae_Sediminibacterium* | 1.42 | *Nocardiaceae_Nocardia* | 1.71 |
| *Alteromonadaceae_Alteromonas* | 1.38 | *Alteromonadaceae_Alteromonas* | 1.61 |
| *Clostridiaceae_1_Clostridium_sensu_stricto_1* | 1.35 | *Corynebacteriaceae_Corynebacterium* | 1.56 |
| *Micrococcaceae_Micrococcaceae_unclassified* | 1.24 | *Oxalobacteraceae_Massilia* | 1.52 |
| *Cytophagaceae_Hymenobacter* | 1.21 | *Bradyrhizobiaceae_Rhodopseudomonas* | 1.41 |
| *Oxalobacteraceae_Massilia* | 1.12 | *Rhodobacteraceae_Paracoccus* | 1.21 |
| *Corynebacteriaceae_Corynebacterium_1* | 1.10 | *Micrococcaceae_Micrococcus* | 1.14 |
| *Rhodobacteraceae_Paracoccus* | 1.03 | *Beijerinckiaceae_Methylocapsa* | 1.10 |
| *Enterobacteriaceae_Enterobacteriaceae_unclassified* | 1.02 | *Pseudomonadaceae_Pseudomonas* | 1.10 |

**Table S6.** Relative abundance of emerging or re-emerging pathogenic bacteria.

| Emerging or re-emerging pathogenic bacteria | Relative abundance in total airborne bacteria（%） |
| --- | --- |
| *Staphylococcus epidermidis* | 0.3408 |
| *Serratia marcescens* | 0.0843 |
| *Corynebacterium amycolatum* | 0.0288 |
| *Aeromonas caviae* | 0.0219 |
| *Staphylococcus aureus* | 0.0154 |
| *Klebsiella pneumoniae* | 0.0036 |
| *Pseudomonas aeruginosa* | 0.0030 |
| *Enterococcus faecalis* | 0.0023 |
| *Aeromonas veronii* | 0.0021 |
| *Campylobacter jejuni* | 0.0021 |
| *Haemophilus influenzae* | 0.0020 |
| *Aeromonas hydrophila* | 0.0012 |
| *Mycobacterium haemophilum* | 0.0008 |
| *Mycobacterium marinum* | 0.0004 |
| *Yersinia enterocolitica* | 0.0003 |
| *Legionella pneumophila* | 0.0003 |
| *Escherichia coli* | 0.0003 |
| *Campylobacter fetus* | 0.0001 |

**Table S7.** The ANOSIM test of pathogenic bacterial communities between different influencing factors.

| Type | Factors | *R* | *P* |
| --- | --- | --- | --- |
| Indoor | People | 0.4219 | **0.004** |
|  | Space | 0.2898 | **0.027** |
|  | Ventilation condition | 0.0828 | 0.289 |
|  | Furnishings | 0.0501 | 0.294 |
| Outdoor | Ground type | 0.1556 | 0.093 |
|  | People | 0.1556 | 0.096 |

**Table S8.** Source and potential health hazards of dominant airborne pathogenic bacteria.

| Pathogenic bacteria | Sources and potential health hazards |
| --- | --- |
| *Saccharopolyspora rectivirgula* | From soil, plants, etc. (Sayed et al., 2020). Causes exogenous allergic alveolitis (Schäfer et al., 2011), "farmer's lung disease" (Barrera et al., 2014) and other diseases. |
| *Acinetobacter johnsonii* | Widely distributed in water, soil, plants, and animals, a common component of the food spoilage flora (Jia et al., 2022), it can cause bloodstream infections, peritonitis, and other diseases (Rodríguez et al., 2014). |
| *Moraxella osloensis* | It causes invasive infections mainly in immunocompromised individuals and can cause bacteremia, septic chest, meningitis and other diseases (Koleri et al., 2022). |
| *Acinetobacter lwoffii* | from food spoilage flora, sewage, etc., which can cause sepsis, pneumonia, meningitis, urinary tract infections, skin and wound infections (Regalado et al., 2009; Rodríguez et al., 2017). |
| *Staphylococcus epidermidis* | It is parasitic in human skin and mucous membranes and can cause skin soft tissue infections, internal organ infections, Staphylococcus epidermidis sepsis (Kleinschmidt et al., 2015), peritonitis, and endocarditis (Brown et al., 2020). |
| *Aerococcus viridans* | It is widely distributed in environments such as hospitals and can cause bacteremia, endocarditis, and urinary tract infections (Mohan et al., 2017) |


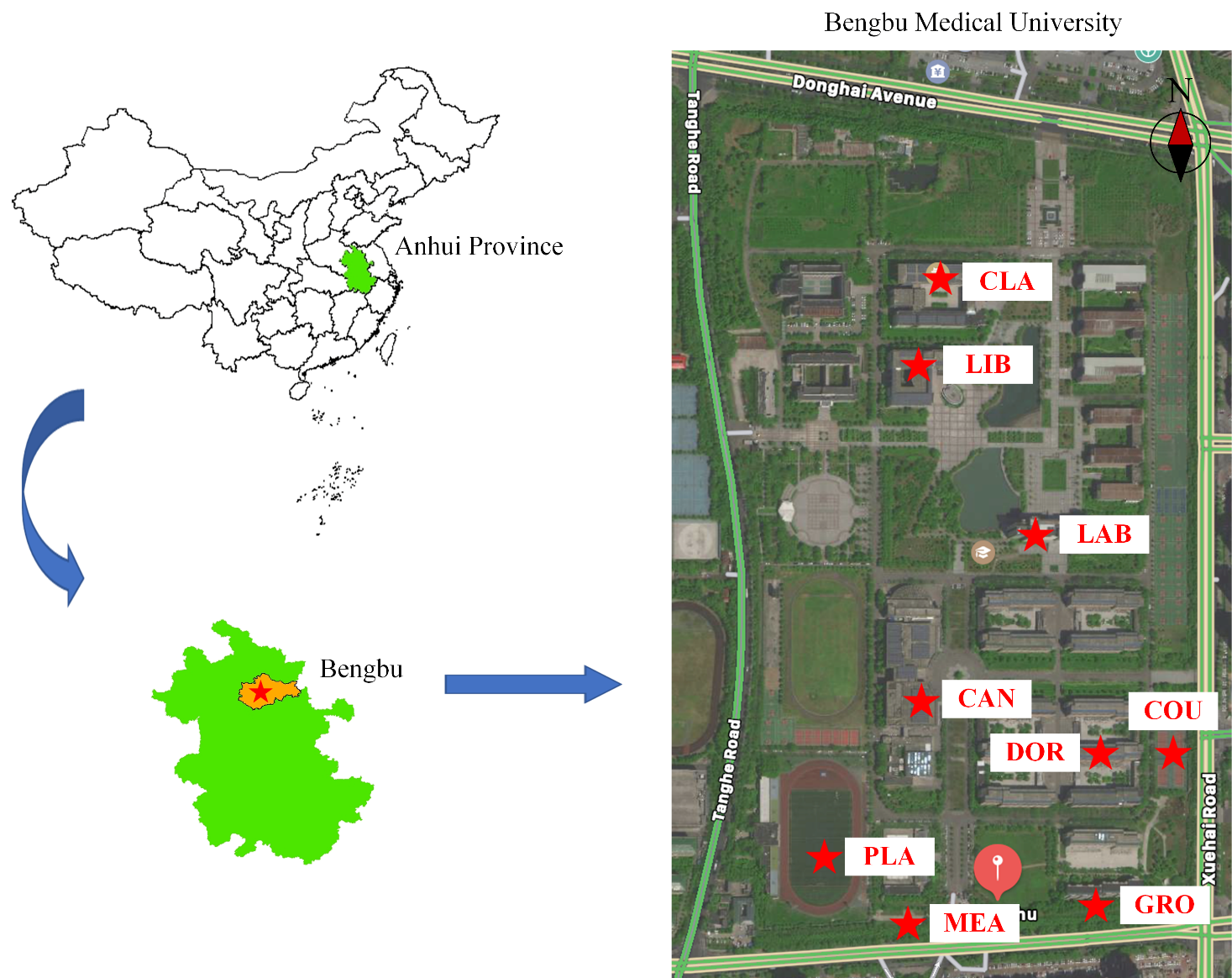


**Figure S1.** Detailed schematic diagram of each sampling point.


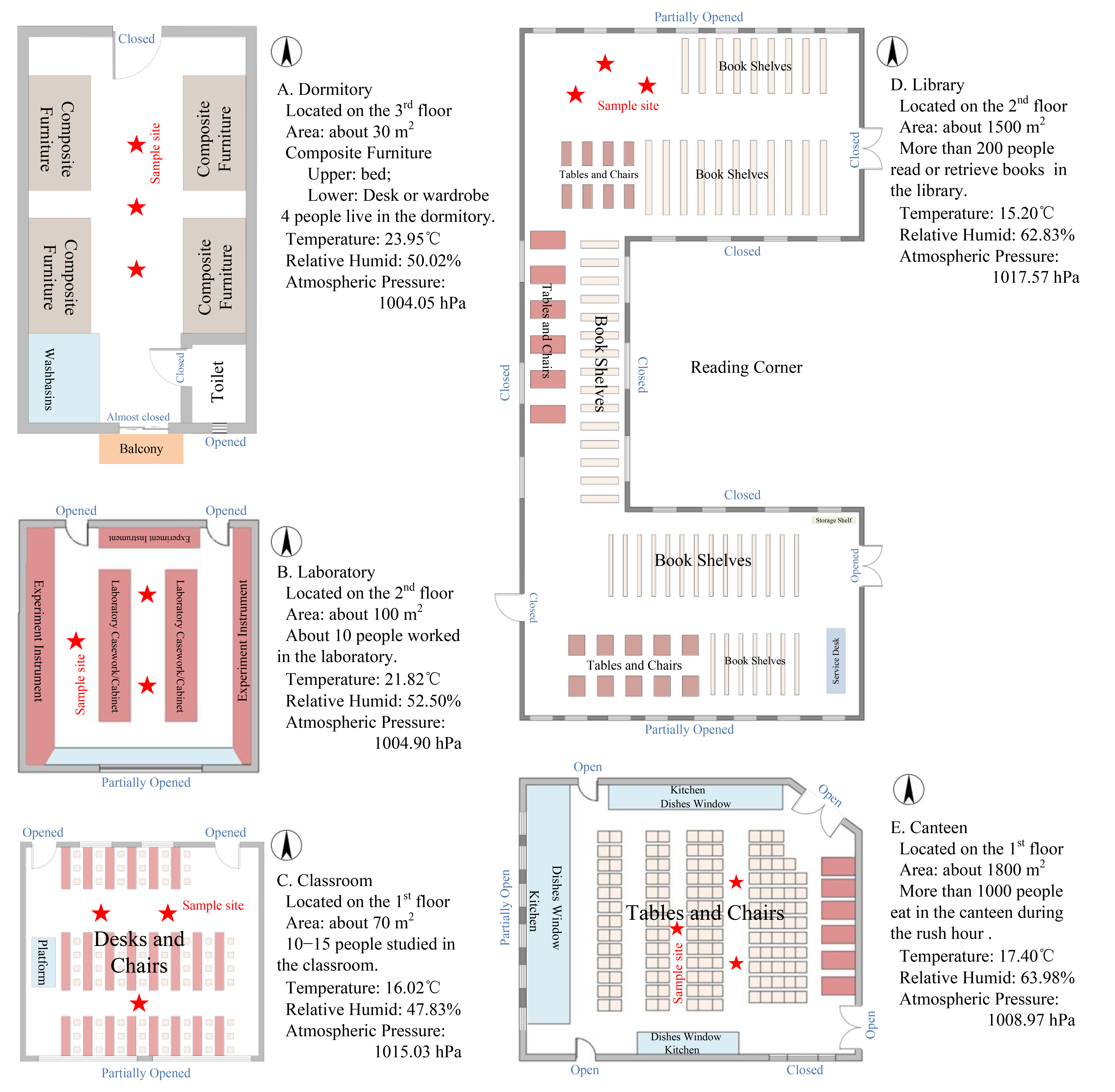


**Figure S2.** Layouts of the five indoor locations.

Note: In the indoor locations, the floors are clad with ceramic tiles without any carpeting. The sampling was conducted in April and the temperature was comfortable. No heating or cooling devices such as air conditioning were used indoors. The rooms were naturally ventilated by window and door openings. There was no intervention in opening or closing on doors and windows. The ventilation conditions which we described as "general" or "better" were based on the degree of the window and door openings.


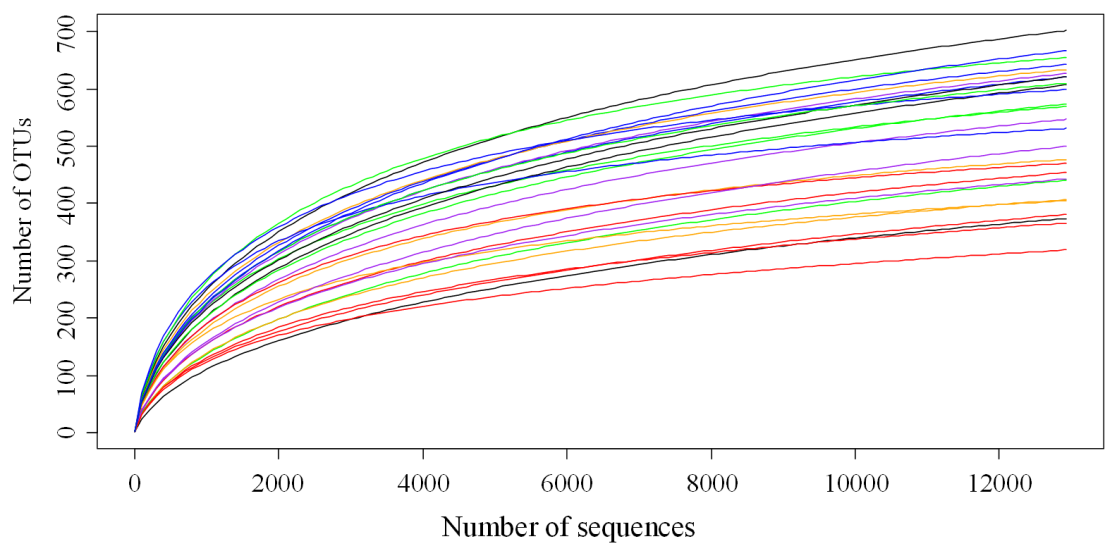


**Figure S3.** The rarefaction curves of campus airborne bacteria at the genus level.


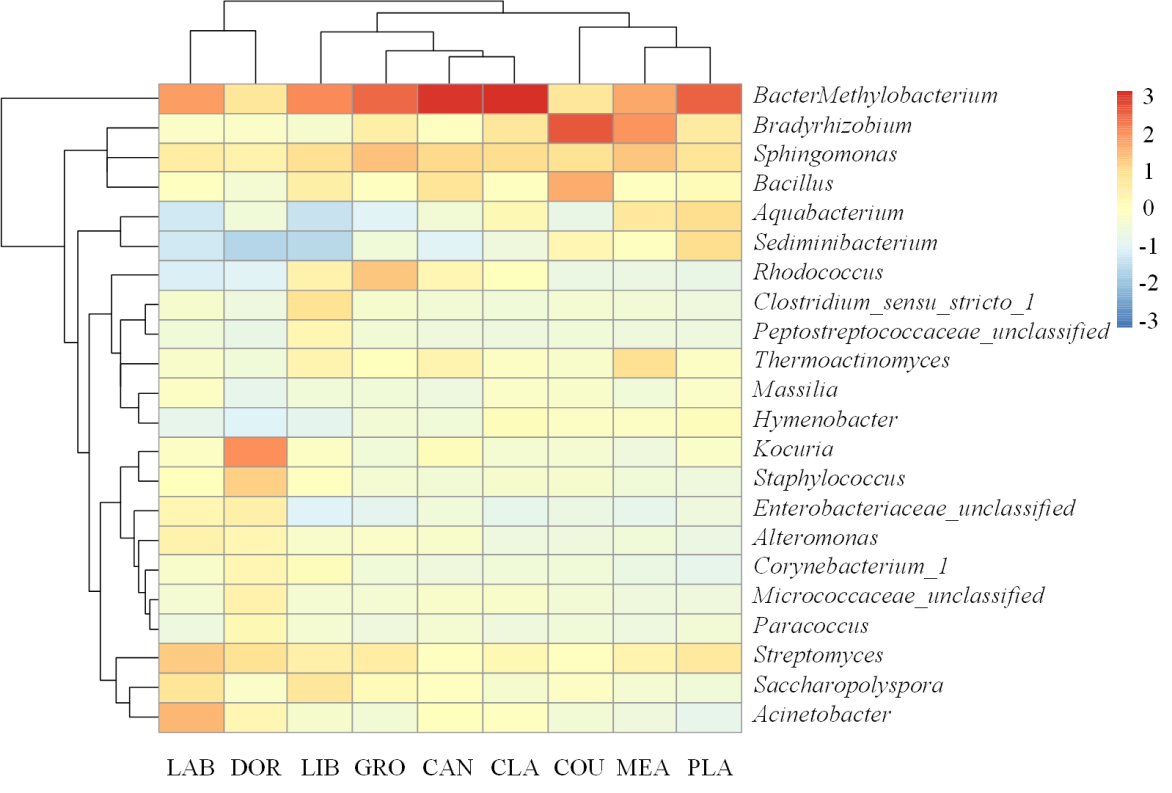


**Figure S4.** Heatmap of the total airborne bacterial communities at different sites.


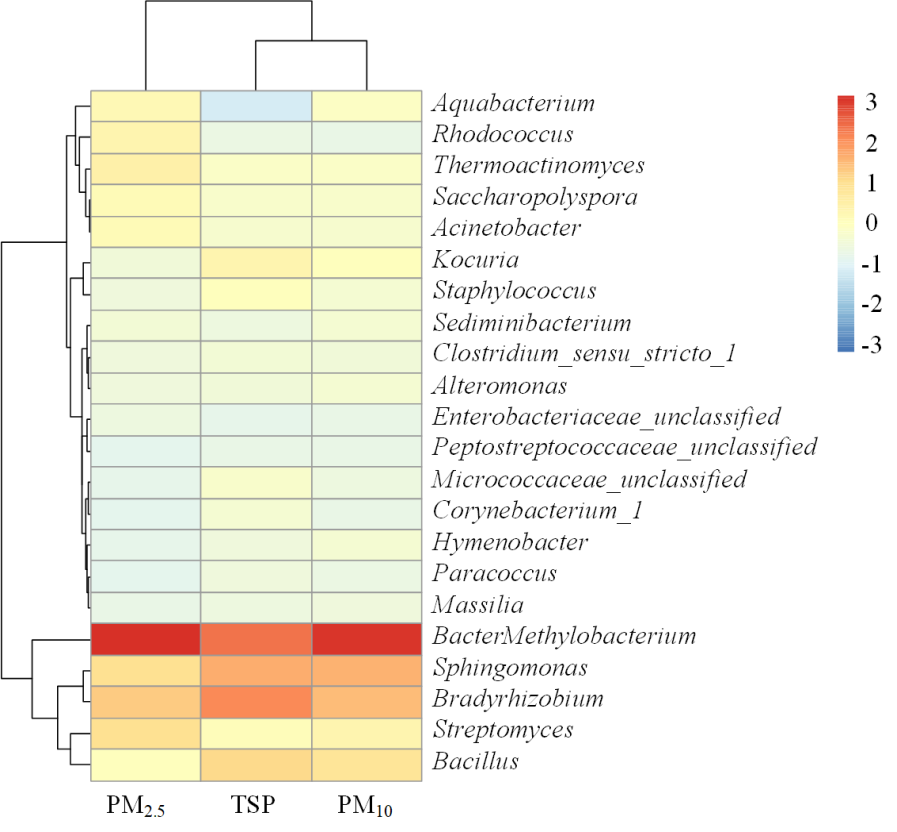


**Figure S5.** Heatmap of the total airborne bacterial communities at different sizes.


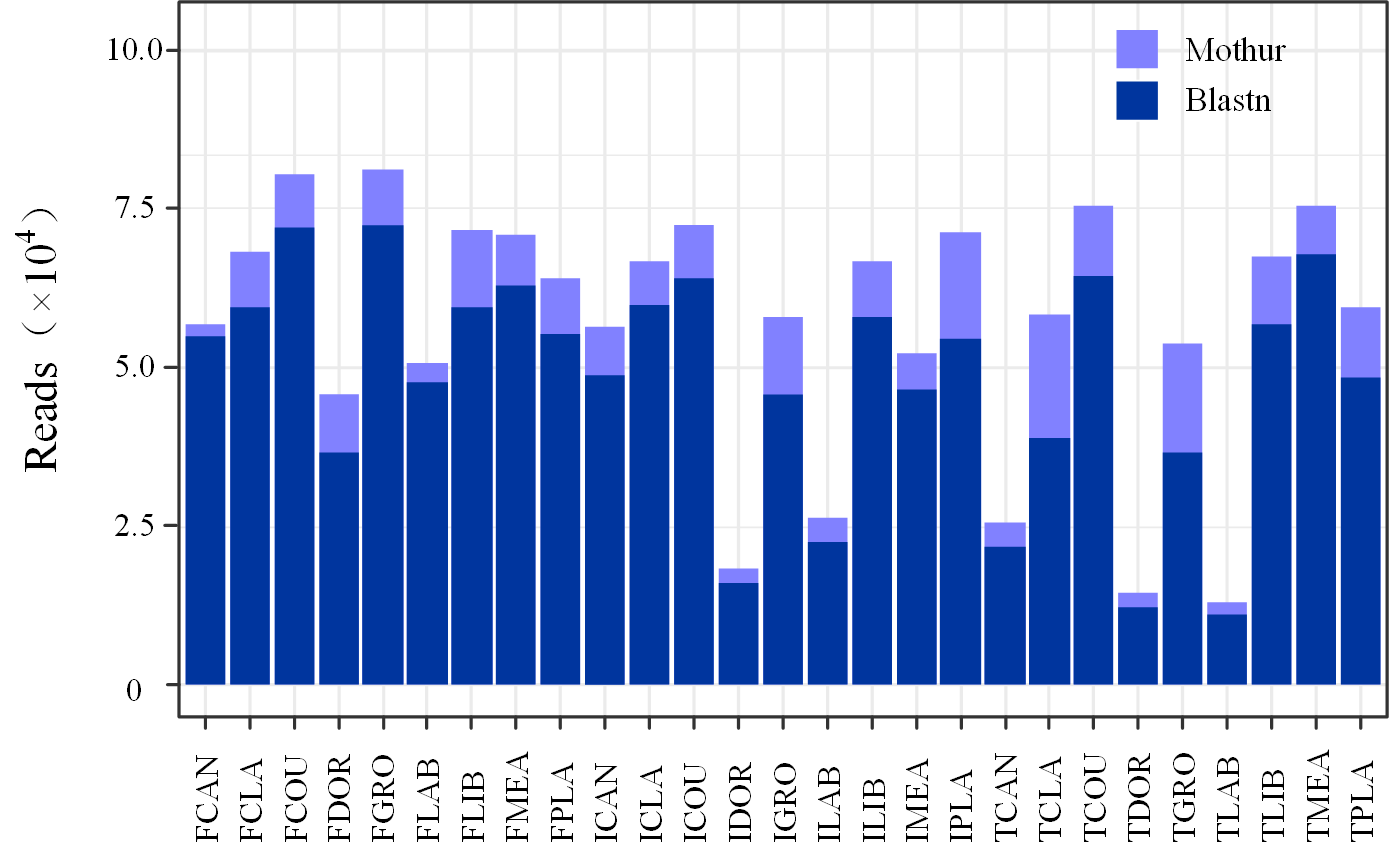


**Figure S6.** The number of airborne bacterial sequences annotated by Mothur analysis and Blastn analysis.


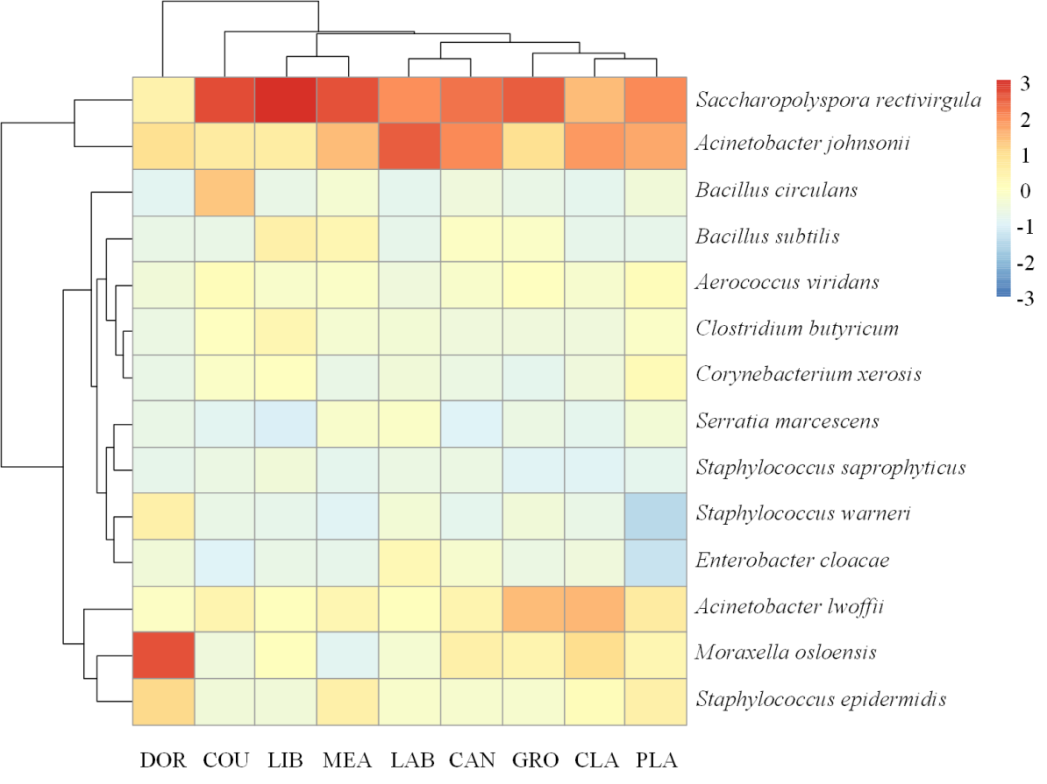


**Figure S7.** Heatmap of pathogenic bacteria at different sites.


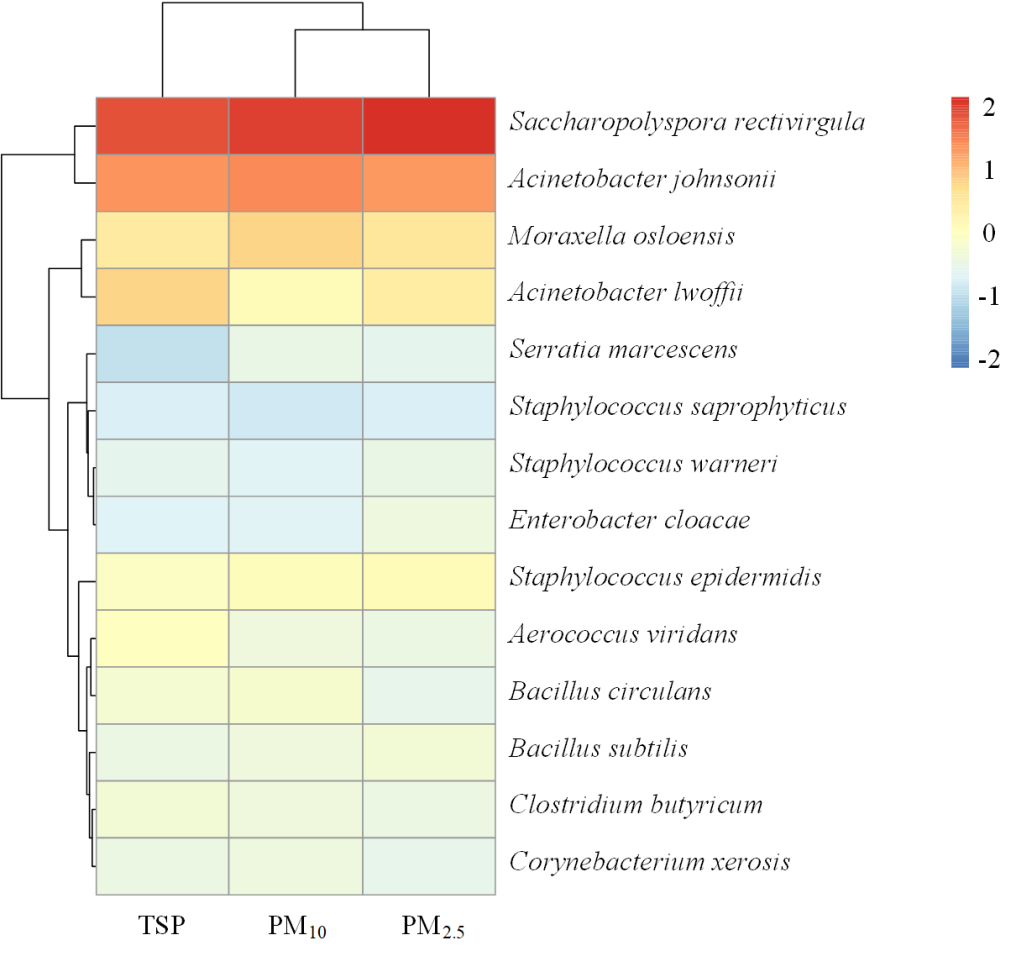


**Figure S8.** Heatmap of pathogenic bacteria at different sizes.

**References**

Barrera, C., Millon, L., Rognon, B., Quadroni, M., Roussel, S., Dalphin, J. C., et al. (2014). Immunoreactive proteins of Saccharopolyspora rectivirgula for farmer's lung serodiagnosis. *Proteomics Clin Appl*. 8, 971-981. doi: 10.1002/prca.201400024

Brown, M. M. and Horswill, A. R. (2020). Staphylococcus epidermidis-Skin friend or foe? *PLoS Pathog*. 16, e1009026. doi: 10.1371/journal.ppat.1009026

Jia, J., Liu, M., Feng, L. and Wang, Z. (2022). Comparative genomic analysis reveals the evolution and environmental adaptation of Acinetobacter johnsonii. *Gene*. 808, 145985. doi: 10.1016/j.gene.2021.145985

Kleinschmidt, S., Huygens, F., Faoagali, J., Rathnayake, I. U. and Hafner, L. M. (2015). Staphylococcus epidermidis as a cause of bacteremia. *Future Microbiol*. 10, 1859-1879. doi: 10.2217/fmb.15.98

Koleri, J., Petkar, H. M., Husain, A. A. M., Almaslamani, M. A. and Omrani, A. S. (2022). Moraxella osloensis bacteremia, a case series and review of the literature. *IDCases*. 27, e01450. doi: 10.1016/j.idcr.2022.e01450

Mohan, B., Zaman, K., Anand, N. and Taneja, N. (2017). Aerococcus Viridans: A Rare Pathogen Causing Urinary Tract Infection. *J Clin Diagn Res*. 11, Dr01-dr03. doi: 10.7860/jcdr/2017/23997.9229

Regalado, N. G., Martin, G. and Antony, S. J. (2009). Acinetobacter lwoffii: bacteremia associated with acute gastroenteritis. *Travel Med Infect Dis*. 7, 316-317. doi: 10.1016/j.tmaid.2009.06.001

Rodríguez, C. H., Nastro, M., Dabos, L., Barberis, C., Vay, C. and Famiglietti, A. (2014). First isolation of Acinetobacter johnsonii co-producing PER-2 and OXA-58 β-lactamases. *Diagn Micr Infec Dis*. 80, 341-342. doi: 10.1016/j.diagmicrobio.2014.09.013

Rodríguez, J. M. and Fernández, L. (2017). Prebiotics and Probiotics in Human Milk. San Diego: Academic Press.

Sayed, A. M., Abdel‐Wahab, N. M., Hassan, H. M. and Abdelmohsen, U. R. (2020). Saccharopolyspora: an underexplored source for bioactive natural products. *J Appl Microbiol*. 128, 314-329. doi: 10.1111/jam.14360

Schäfer, J., Kämpfer, P. and Jäckel, U. (2011). Detection of Saccharopolyspora rectivirgula by quantitative real-time PCR. *Ann Occup Hyg*. 55, 612-619. doi: 10.1093/annhyg/mer018
